# Bilateral Single-Port Sympathectomy: Long-Term Results and Quality of Life

**DOI:** 10.1155/2013/348017

**Published:** 2013-12-08

**Authors:** Mohsen Ibrahim, Cecilia Menna, Claudio Andreetti, Anna Maria Ciccone, Antonio D'Andrilli, Giulio Maurizi, Leda Marina Pomes, Francesco Cassiano, Federico Venuta, Erino A. Rendina

**Affiliations:** ^1^Division of Thoracic Surgery, Sant'Andrea Hospital, Faculty of Medicine and Psychology, University of Rome “Sapienza”, Via di Grottarossa 1035, 00189 Rome, Italy; ^2^Division of Thoracic Surgery, “G. Mazzini” Hospital of Teramo, Faculty of Medicine and Surgery, University of L'Aquila, Piazza Italia 1, 64100 Teramo, Italy; ^3^Division of Thoracic Surgery, Policlinico Umberto I Hospital, Faculty of Medicine and Pharmacy, University of Rome “Sapienza”, Viale del Policlinico 155, 00186 Rome, Italy; ^4^Fondazione Eleonora Lorillard Spencer Cenci, Via Di Casal de' Pazzi 10, 00156 Rome, Italy

## Abstract

*Object*. Video-assisted thoracoscopic sympathectomy is a safe, effective, and minimally invasive procedure for primary hyperhidrosis. This study aims to evaluate long-term results and patients' quality of life and investigate potential variables responsible for compensatory sweating after one-stage bilateral single-port thoracoscopic sympathectomy. *Methods*. Between 2005 and 2011, 260 consecutive bilateral thoracoscopic sympathectomies were performed in 130 patients for primary palmar and axillary hyperidrosis through one-port access. Residual pain, postoperative complications, recurrence of symptoms, heart rate adjustment, and quality of life were analyzed. Multivariate analysis was performed. *Results*. No operative mortality and conversion to open surgery were recorded. Mean operative time was 38 ± 5 minutes. Mean hospital stay was 1.1 ± 0.6 days. Eight patients (6%) had unilateral pneumothorax. Twenty-five cases (19%) were complicated by compensatory sweating. Winter and fall were identified as protective factors for compensatory sweating occurrence. Decreased heart rate was observed 1 year after surgery and permanently over the time. No recurrence during the follow-up period (31.5 months) was observed and 90% of patients showed improved quality of life. *Conclusions*. One-stage bilateral miniuniportal thoracoscopic sympathectomy is a valid and safe treatment for primary hyperhidrosis, achieving definitive and esthetic results, with excellent patients' satisfaction. Compensatory sweating may potentially occur in a season-dependent manner.

## 1. Introduction

Primary hyperhidrosis is a disorder characterized by excessive perspiration beyond thermoregulatory needs, particularly in response to temperature or emotional stimuli. Primary hyperhidrosis has an estimated prevalence of nearly 3% [1]. Severe hyperhidrosis commonly affects hands, face, axillae, and feet, causing significant medical and psychosocial consequences. Medical treatments, as local antiperspirants, systemic anticholinergic agents, iontophoresis, and botulinum toxin, alleviate symptoms only transiently. However, surgical therapy is the most effective and recognized as the treatment of choice for patients with primary hyperhidrosis.

To date among all the different surgical approaches, video-assisted thoracoscopic sympathectomy has been shown as safe and minimally invasive procedure for palmar and axillary hyperhidrosis [2]. In addition, it can be performed using single or multiple ports [3, 4]. Both uniportal and biportal video-assisted thoracoscopic sympathectomy have been demonstrated to be effective, safe, and minimally invasive for palmar hyperhidrosis. Comparing with the biportal approach, the uniportal approach causes less postoperative pain and less operative time [5], and it consists in a more reasonable procedure in treatment of palmar hyperhidrosis in terms of esthetic results [6].

The present study aims to show (1) the long-term results, (2) the quality of life (QoL) improvement, and (3) potential variables responsible for compensatory sweating after bilateral single-port thoracoscopic sympathectomy in 130 treated patients.

## 2. Materials and Methods

### 2.1. Patients

Between April 2005 and February 2011 in the Thoracic Surgery Department, Sant'Andrea Hospital, Rome, 130 patients underwent 260 consecutive video-assisted thoracoscopic sympathectomies through one port access for primary palmar and axillary hyperidrosis. All patients had excessive sweating in hands and armpits, severely interfering with their work or social activities. The clinical characteristics of patients are listed in Table 1. Before surgery, all patients underwent a careful clinical history, preoperative routine blood examination, spirometry, cardiological consulting with heart rate detection, and chest X-ray to exclude pulmonary affections. Patients with secondary hyperhidrosis were excluded from this study. A written informed consent from all patients and a study approval from Ethics Committee were obtained before surgery.

The mean postoperative follow-up period was 31.5 months (range: 1–72 months). Heart rate, compensatory sweating, and recurrence of symptoms were evaluated one year after surgery and every year after. Recurrence of symptoms was assessed by a quantitative sudomotor axon reflex. In addition, QoL was assessed administering a standardized study questionnaire used by De Campos et al. [7] and suggested by the Society of Thoracic Surgeons Expert Consensus for the Surgical Treatment of Hyperhidrosis [8]. The questionnaire, shown in Table 2, evaluates 20 items divided into 4 domains (functional, personal, emotional, under special circumstances domains), rating the QoL from one (excellent) to five (very poor). The minimum score (20) indicates an excellent QoL and the maximum score (100) indicates a very poor QoL. QoL was assessed before and one year after surgery.

### 2.2. Surgical Technique

Surgery was performed by the same equipe under general anesthesia. Double-lumen endotracheal intubation and selective one-lung ventilation were used. Patients were placed on the operating table in a semisitting position with arms abducted more than 90°. Only one incision of 8 mm was performed in the third intercostal space on the anterior axillary line. The procedure was performed in the same body position on the other side.

Three mm, 30° thoracoscope (KARL STORZ, Tuttlingen, Germany), and 5 mm endoscopic dissector (B. BRAUN, Melsungen, Germany) were inserted into the thoracic cavity after lung exclusion on the operative side of surgery. The sympathetic chain was identified running down over the necks of the ribs. By opening the parietal pleura, the sympathetic chain was exposed, recognizing the T2-T4 tract. Dissection was performed by electrocautery from the second to the fourth ganglia. Then, the thoracoscope and endoscopic instruments were removed. A temporary 10 Ch chest tube was inserted into the thoracic cavity through the previous incision and connected to a water seal system applying a mild suction, while the procedure was completed on the other side. After reinflating the lungs under direct vision, the chest tubes were quickly removed and the incisions were closed on each side. All specimens obtained from dissected sympathetic chain were analyzed by histology. Chest X-ray was performed during the first postoperative day before the discharge. Characteristics of patients and clinical data as operating time, hospital stay, residual pain, postoperative complications, recurrence of symptoms, and patient satisfaction were analyzed.

### 2.3. Statistical Analysis

Values are reported as means ± standard deviation. Comparisons between preoperative and postoperative values of heart rate and quality of life scores were analyzed using paired *t*-test as all variables were normally distributed. Potential variables responsible for compensatory sweating and postoperative pneumothorax, as age (≤30 years/≥31 years), gender, smoking status, and seasons (when the surgical procedure was performed) were evaluated by logistic regression analysis. The odds ratio (OD) and corresponding 95% confidence intervals were reported for covariates, considering clinically relevant the .05 significance level.

## 3. Results 

All patients had an immediate beneficial effect after surgery, showing warm and dry hands, as well as full satisfaction. Histology analysis confirmed a normal nervous tissue.

A total of 260 video-assisted thoracoscopic sympathectomies were performed in 130 patients. No operative mortality neither conversion to open surgery were recorded (Table 3). The mean operative time was 38 ± 5.0 minutes. The mean hospital stay was 1.136 ± 0.6 days.

**Figure 1:**
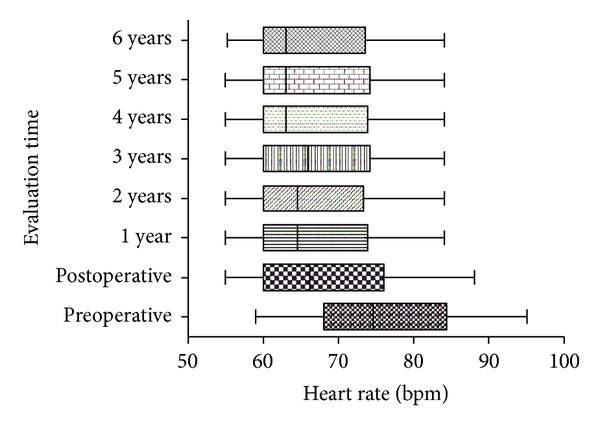
Heart rate adjustments during the follow-up period.

Within 7 days after operation, 16 patients (12%) suffered from mild/moderate pain, requiring more analgesics than the standard doses. After 7 days following surgery, no cases of residual pain were recorded. Eight patients (6%) had a unilateral pneumothorax after surgery. One of them (0,3%) required positioning of chest drainage; the others were treated by rest and respiratory physiotherapy. Patients with pneumothorax were discharged after 4 days. No patient experienced Horner's syndrome. Twenty-five cases (19%) were complicated by compensatory sweating. According to the logistic regression analysis (Table 4), winter and fall seasons (when surgery was performed) were identified as protective factor for compensatory sweating occurrence (OR = 0.21, *P* = 0.01). Preoperative heart rate mean values decreased from 77 ± 10.6 bpm (beats per minute) to 69 ± 8.7 bpm in the first postoperative day (*P* < 0.001), remaining stable over the follow-up period (Figure 1). No recurrence of symptoms was observed during the follow-up period, demonstrated by a negative quantitative sudomotor axon reflex result. Resolution of palmar hyperhidrosis was complete in 100% of patients. An improvement in postoperative QoL scores was observed in 90% of patients at one year after surgery (35 ± 15 versus 90 ± 12, *P* < 0.001).

## 4. Discussion

Before the introduction of video-assisted thoracoscopic surgery (VATS) and the advances in video-endoscopic technology, thoracotomy was the standard surgical approach for hyperhidrosis [9–11]. VATS had replaced open surgery to perform sympathectomy, determining a shorter hospital stay, reduced morbidity rates, less pain, and better cosmetic results for a non-life-risk disease [12, 13]. Primary hyperhidrosis negatively seems to affect the following areas of the life: work (88%), friendships (73%), relations with partner (46%), and family (21%). Indeed, this negative repercussion motivates patients to undergo surgery to solve the problem [14]. The history of uniportal VATS stretches back almost a decade with the treatment of simple thoracic conditions. As the technique matures with increasing ability to tackle the full spectrum of thoracic surgical diseases, most notably major lung resections for lung tumours, the spread of uniportal VATS across the globe has been phenomenal. VATS centres all over the world are now performing uniportal VATS and developing their individual styles and techniques with great successes. In this report we have shown that one-stage bilateral video-assisted thoracoscopic sympathectomy for palmar and axillary hyperhidrosis carried out through a single port can be a successful and riskless procedure with minimal invasiveness and slight postoperative pain. Moreover, we showed that the single-port thoracoscopic sympathectomy technique is equally feasible as multiple ports.

The mean operating time was quite short (38 ± 5.0 minutes). We did not need to reposition the patient to operate on the other side. In line with other studies reporting the use of single port procedure [15], the mean hospital stay was 1.136 ± 0.6 days.

The results are conditioned by the surgical technique used. Interruption of the chain can be achieved by cauterizing, cutting, or clipping the sympathetic chain. The level of interruption remains controversial. Although an expert consensus exists and provides standardized suggested treatment strategies [8], there remains some controversy. In our experience, surgical ablation of the T2-T4 ganglia had excellent results: the target resolution of the disorder was achieved in 100% of the patients. No recurrence was observed. These results are in line with those reported by other authors [16]. No mortality was described in our experience. Previous studies showed that the overall intraoperative morbidity (i.e. chylothorax, lung, or vessels damage) is nearly 0.2% [14], reporting complications during surgery and conversion to thoracotomy [17, 18]. However, none of these issues was observed in our study. Pneumothorax was the most common early complication (6% of patients), although only 0,3% of cases required pleural drainage, according to data reported in the literature (less than 10%) [19]. However, an exertion of continuous positive pressure for a few seconds in coordination with the anesthesiologist during the suture of the skin and the application of a mild suction to the temporary chest tube during the other side procedure are essential to prevent residual air and possible incomplete reexpansion of the lung [20, 21]. However, as in all thoracoscopic procedures, it is quite common to find air in the thoracic cavity at the end of surgery and for few days later. Postoperative pain lasting less than 1 week was observed in 12% of our cases: only 16 patients required morphinics or local analgesia with naropin infiltration in addition to the standard doses of analgesics (90 mg of ketorolac and 150 mg tramadol hydrochloride). There was no significant relevance for constant residual pain after 7 days following operation. Postoperative pain after video-assisted thoracoscopic sympathectomy has been previously analyzed by De Campos et al. [19]. No significant association was found between the type of scalpel used and the severity of the pain. There was no difference between harmonic and electric scalpel use in the levels of thoracic pain during the first 30 days after VATS. Pain symptoms are mostly related to trauma of the thoracic wall caused while introducing the trocars into the intercostal space and periosteal lesions close to the rib head. Lesions caused by transmission of heat from the electric scalpel to tissues close to the sympathetic chain are minimal and not significant to produce an increased pain score.

We confirmed that the use of uniportal VATS to perform sympathectomy allows us to achieve lower morbidity rates and satisfying postoperative outcomes [22–24]. Compensatory hyperhidrosis (postoperative increase of sweating in regions of the body where it had not been previously observed) is the most common late complication, with different incidence reported in previous studies, ranging from 33% to 85% [25–27], regardless of the number of ganglia removed [28], showing a gradually decreasing intensity over the follow-up period. However, the mechanism of compensatory hyperhidrosis is still unclear. An alternative to reduce compensatory sweating consists in ablating only one ganglion, being the lowest (T4) and the best alternative. Otherwise, applying metal clips to interrupt the sympathetic chain by compression can affect the postoperative compensatory sweating occurrence [29]. In our study single-port bilateral thoracoscopic sympathectomy did not rule out the occurrence of a compensatory hyperhidrosis (19% of cases), although it is mainly moderate and well tolerated by patients. Not all studies assess compensatory sweating or identify variables related to the occurrence of the main side effect after surgical procedure. We investigated if variables as age, weather factors, gender, and smoking habits could impact on compensatory sweating incidence. In this report we demonstrated that all the above-mentioned variables but weather did not affect compensatory sweating. Specifically, we provided for the first time a possible correlation between a colder seasonal change (fall and winter) and a positive effect on the compensatory sweating occurrence.

We can speculate that although the sudomotor function remains intact in these patients thus the threshold of metabolic heat production evaporation, however the sympathectomy leads to an imbalance of these factors, exacerbated by warmer seasons (summer) when the increased sweating occurs per se [30]. Therefore, patients should be always fully informed about both the possibility of compensatory sweating and the seasonal correlation.

In addition, we provided a detailed report on long-term results and QoL after one-stage single-port bilateral VATS sympathectomy. It is noteworthy to mention that 10% of patients did not improve the QoL because of compensatory sweating effect. In contrast, 9% of patients who experienced mild/moderate compensatory sweating significantly improved their QoL.

A standardized data collection is needed to quantify patients' QoL. A worldwide adoption of a uniformed anatomic approach to VATS sympathectomy, as well as systematic assessments of results and QoL improvement, allows advancing toward evidence-based clinical practice.

## 5. Conclusions

In conclusion, our results suggest that one-stage miniuniportal bilateral video-assisted thoracoscopic sympathectomy is a valid and secure treatment for palmar and axillary hyperhidrosis. This procedure is feasible, achieving definitive and esthetic results, with minimally invasive technique and excellent patient's satisfaction. In clinical practice, patients should be informed that their QoL may definitely improve after a uniportal procedure, unless compensatory sweating occurs. Compensatory sweating occurrence can arise in a season-dependent manner.

## Figures and Tables

**Table 1:** Clinical characteristics of patients.

Variables	*n*	%
Sex (male/female)	49/81	38/62
Mean age/range/SD (years)	32,04/16–63/11,34	—
Family history (yes/no)	23/107	18/82
Previous lung disease (yes/no)	0/130	0/100
Previous treatments (yes/no)	104/26	80/20
Palmar hyperhidrosis (yes/no)	130/0	100/0
Axillary hyperhidrosis (yes/no)	91/39	70/30

SD: standard deviation.

**Table 2:** Quality of life questionnaire (administered preoperatively and postoperatively).

Generally speaking, how would you rate your quality of life *currently*?					
L: excellent 2: very good 3: good 4: poor/inferior 5: very poor					
Using the same scale as above (1–5), how would you rate the following activities currently?					

Writing	1	2	3	4	5
Manual work	1	2	3	4	5
Leisure	1	2	3	4	5
Sports	1	2	3	4	5
Hand shaking	1	2	3	4	5
Socializing	1	2	3	4	5
Grasping objects	1	2	3	4	5
Social dancing	1	2	3	4	5

Personal domain: with partner/spouse, how would you rate your quality of life?					

Holding hands	1	2	3	4	5
Intimate touching	1	2	3	4	5
Intimate affairs	1	2	3	4	5

Emotional self/others: how would you rate the fact that after sweating excessively?					

I have always justified myself	1	2	3	4	5
People rejected me slightly	1	2	3	4	5

Under special circumstances: how would you rate the quality of your life?					

In a closed or hot environment	1	2	3	4	5
When tense or worried	1	2	3	4	5
Thinking about the problem	1	2	3	4	5
Before a test, meeting, or public speaking	1	2	3	4	5
Wearing sandals/barefoot	1	2	3	4	5
Wearing colored clothing	1	2	3	4	5
Having problems at school/work	1	2	3	4	5

**Table 3:** Results.

Variables	*n*
Winter/fall* (*n*/*n*)	56/21
Summer/spring* (*n*/*n*)	40/13
Mean operative time (min)	38.0 ± 5.0
Mean hospital stay (days)	1.1 ± 0.6
Residual pain in 1 week (*n*/%)	16/12
Pneumothorax (*n*/%)	8/6
Compensatory sweating (*n*/%)	25/19
Horner's syndrome (*n*/%)	0/0
Chylothorox (*n*/%)	0/0
Recurrence (*n*/%)	0/0

*When the surgical procedure was performed.

**Table 4:** Logistic regression analysis of compensatory sweating.

Variables	OR	*P* value	95% confidence interval
Season*	0.21	0.01	0.13–0.84
Smoking status	1.31	0.49	0.59–2.89
Gender	0.74	0.45	0.33–1.63
Age	1.28	0.41	1.16–9.05
Cons	0.09	0.00	0.31-0.30

OR: odds ratio.

*Winter/fall versus summer/spring.
